# Skin Layer Thickness and Shear Wave Elastography Changes Induced by Intensive Decongestive Treatment of Lower Limb Lymphedema

**DOI:** 10.1089/lrb.2021.0036

**Published:** 2022-02-28

**Authors:** Merriem Zarrad, Claire Duflos, Gregory Marin, Murielle Benhamou, Jean-Pierre Laroche, Michel Dauzat, Isabelle Quéré, Sandrine Mestre-Godin

**Affiliations:** ^1^ Vascular Medicine Department, Montpellier University Hospital, Montpellier, France.; ^2^ Clinical Research and Epidemiology Unit, Medical Information Department, Montpellier University Hospital, Montpellier, France.; ^3^ Vascular Medicine Department, Lymphology Unit, Montpellier University Hospital, Montpellier, France.; ^4^ Vascular Medicine, Nimes University Hospital, Nimes, France.; ^5^ Desbrest Institute of Epidemiology and Public Health (IDESP), INSERM—Montpellier University, Montpellier, France.

**Keywords:** lymphedema, lower limb, intensive decongestive therapy, B-mode ultrasonography, elastography

## Abstract

***Background:*** A detailed quantitative evaluation would be beneficial for management of patients with limb lymphedema.

***Methods and Results:*** In 47 patients with lower limb lymphedema at International Society of Lymphology clinical stage 2A (18 limbs), 2B (41 limbs), and 3 (13 limbs), we measured the limb circumference and thickness of epidermis, dermis, and subcutis layers with B-mode ultrasonography and subcutis elastic modulus with ultrafast shear wave velocity (ultrasound elastography) at 5 anatomical levels (M1 to M5) before and after a 3- to 5-day intensive decongestive therapy (IDT) session. Limb circumference and thickness of the epidermis, dermis, and subcutis were greater in the 72 limbs with lymphedema than in the 22 unaffected limbs before and after IDT. The affected limb volume was 10,980 [8458–13,960] mL before and 9607 [7720–11,830] mL after IDT (*p* < 0.0001). The IDT-induced change in subcutis thickness was −9 [−25 to 13]% (NS), −11 [−26 to 3]% (*p* = 0.001), −18 [−40 to −1]% (*p* < 0.0001), −15 [−35 to 3]% (*p* = 0.0003), and −25 [−45 to −4]% (*p* < 0.0001) and significantly correlated with the change in elastic modulus, which was 13 [−21 to 90]% (*p* = 0.004), 33 [−27 to 115]% (*p* = 0.0002), 40[−13 to 169]% (*p* < 0.0001), 9 [−36 to 157]% (*p* = 0.024), and −13 [−40 to 97]% (NS), respectively, at the M1, M2, M3, M4, and M5 levels. Intraobserver reproducibility was satisfactory for skin thickness and fairly good for elastography, but interobserver reproducibility was poor or unacceptable.

***Conclusions:*** IDT reduced the circumference and subcutis thickness of lower limbs with lymphedema and increased their elastic modulus, implying greater tissue stiffness probably due to fluid evacuation. Although subcutis thickness measurement proved to be reliable, technological and methodological improvements are required before ultrasonographic elastography can be used in clinical practice.

## Introduction

If untreated, lymphedema can become progressively irreversible, with increased skin thickness and stiffness together with adipose tissue hypertrophy and fibrosis.^1,2^ High-resolution B-mode ultrasonography (US) is increasingly used for evaluation of skin thickness and echogenicity in lymphedema^3–6^ and of the effect of intensive decongestive therapy (IDT),^7,8^ but remains hindered by limited sensitivity, specificity,^9^ and reproducibility.^10^

Ultrasound elastography is widely used for biomechanical tissue characterization, especially for liver, thyroid, and breast diseases.^11^ From their literature review, DeJong et al. concluded that evidence supporting the use of ultrasound elastography for the diagnosis of cutaneous conditions remained low, mainly because of small sample sizes and risks of bias.^12^ Forte et al. conducted a literature review focused on the use of elastography for staging of cancer-related lymphedema and concluded that it showed great potential.^13^

The primary aim of the present study was to assess changes in skin thickness and tissue elastic modulus induced by IDT in patients with lower limb lymphedema. The secondary goals were to compare ultrasonographic data between lymphedema stages^14^ and to investigate correlations between changes in elastic modulus, limb circumference, and thickness of skin layers.

## Materials and Methods

### Population sample

This single-center, longitudinal, prospective observational study was performed on inpatients with uni- or bilateral, primary or secondary, stage 2 or 3 lower limb lymphedema^14,15^ referred for IDT.

As per the International Society of Lymphology criteria,^16^ stage 2 was defined as permanent limb swelling not reduced by elevation (2A: pitting sign and preserved skin suppleness; and 2B: reduced pitting sign and increased skin thickness) and stage 3 as permanent limb swelling with hardened tissues, skin changes (e.g., hardened skin and papillomas), and absent pitting sign. Lymphedema diagnosis and staging relied on clinical data and anamnesis in patients with secondary lymphedema and on lymphoscintigraphy in patients with primary lymphedema. This study was approved by the institutional review board (#2019_IRB-MTP_11-01) and each participant provided a written informed consent.

Noninclusion criteria were cardiac, renal, or hepatic disease that could contribute to limb edema; chronic venous disease with reflux or history of venous thrombosis; history of surgery on the affected lower limb; or skin lesions precluding ultrasonographic examination. Pregnant women, patients under 18 years of age, and patients unable or unwilling to provide informed consent were not included.

**FIG. 1.**
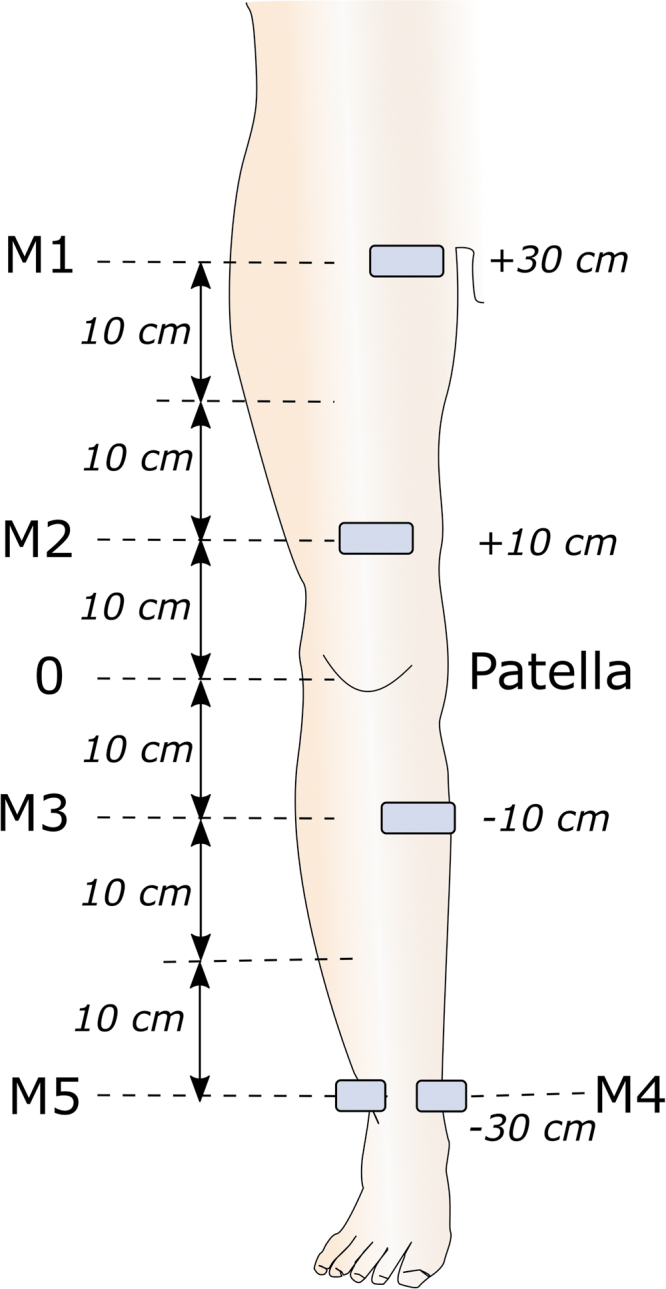
Sites of ultrasonographic measurements.

The ultrasonographic examination was part of the routine evaluation of inpatients at hospital admission and at the end of the IDT session. We measured the body weight, height, and lower limb circumference 30, 20, and 10 cm above the patella; at the patella apex; and 10, 20, and 30 cm below the patella (Fig. 1). The limb volume was then calculated as


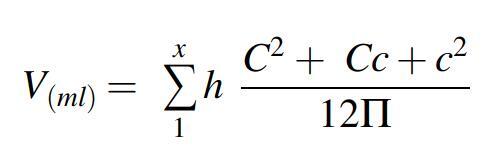


with *C* and *c* = larger and smaller, respectively, limb circumferences (in cm) at each measurement level and *h* = 10 cm.^17^

### Ultrasonographic evaluation

Using an Ultimate ultrasound system (Aixplorer, Aix-en-Provence, France) with its SL10-2 linear probe, we performed measurements on the medial aspect of the thigh at 30 (M1) and 10 cm (M2) above the patella, on the medial aspect of the calf 10 cm below the patella (M3), and on the medial and lateral aspects of the ankle at the malleolus level (M5) (Fig. 1).

**FIG. 2.**
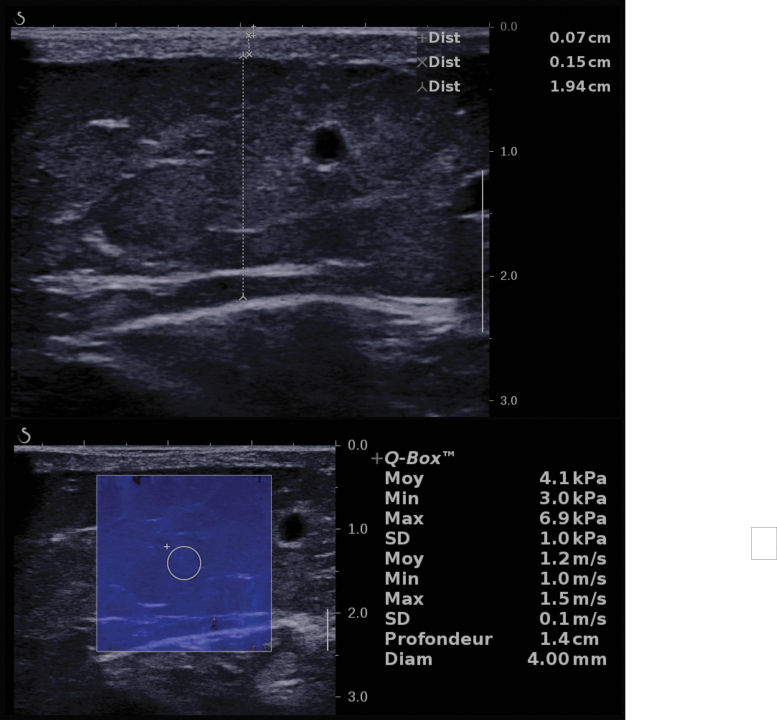
Ultrasonographic measurement of skin layer thickness and elastography. B-mode ultrasonographic image with thickness measurement of skin layers (*upper panel*), and elastography measurement (*lower panel*) in a limb with lymphedema.

Thickness of the epidermis, dermis, and subcutis was measured, respectively, between the probe surface and the epidermis–dermis interface, between the epidermis–dermis interface and the dermis–subcutis interface, and between the dermis–subcutis interface and the muscle aponeurosis (Fig. 2 and Supplementary Data).^18,19^

Elastography was performed using ultrafast shear wave velocity (SWV) imaging (Supplementary Data)^20^ to measure the tissue elastic modulus within a 4-mm circular sample volume at the mid-depth of the subcutis layer, avoiding vessels, aponeuroses, and areas of liquid collection (Fig. 2). The pressure exerted on the patient's skin by the ultrasound probe was measured with a XFTC300 sensor and ARD154 amplifier (Measurement Specialties, Hampton, VA)^21^ and displayed on a personal computer so that the operator could apply minimal force on the probe during elastography measurement.

### Intensive decongestive therapy

IDT included skin care and hydration, manual lymphatic drainage, personalized multilayer compression, gymnastics when wearing the compression garment, and self-therapy training.^22,23^ Compression was achieved with inelastic bandaging generating an interface pressure greater than 45 mm Hg. Limb status and volume were reevaluated and compression devices were replaced and adjusted every day.^15^ For patients with stage 2 and 3 lymphedema, the first two IDT sessions lasted 5 days. Subsequent sessions lasted 3 days for patients with stage 2 lymphedema and for patients with stage 3 if the limb volume excess was under 20% or if the delay from the last session was under 1 year.

### Statistical analyses

Before the present study, we performed a feasibility study (data not published), which showed that Cohen's d (effect size of IDT-induced elastic modulus change) ranged from 0.57 to 1.12 at the M3 and M4 levels. Therefore, we calculated that 44 patients were needed to be able to obtain an effect size of 0.7 with a power of 90% and significance level of 0.005. This low *p*-value was chosen because of the large number of tests performed for the principal analysis. Categorical data are reported as number (percentage) and compared using the Fisher exact test with Freeman–Halton extension when appropriate. The distribution of variables was assessed with the D'Agostino and Pearson omnibus normality test. Quantitative variables are reported as mean ± standard deviation (SD) if normally distributed and median [lower–upper quartile] if not.

Differences between two groups (independent data) and changes within one group (paired data) were evaluated with Student's t test if normally distributed and the Wilcoxon–Mann–Whitney test and Wilcoxon signed-rank test if not. Comparisons between lymphedema stages were performed with the Kruskal–Wallis test, followed by Dunn's multiple comparison test. Values of *p* < 0.05 were considered significant for all tests except the principal analysis (*p* < 0.005). Correlations between continuous variables were investigated using the Spearman r coefficient.

Reproducibility was assessed by the Lin concordance correlation coefficient (CCC) with two-sided confidence intervals in 30 patients (30 limbs) for intraobserver reproducibility and 40 patients (40 limbs) for interobserver reproducibility. Reproducibility was considered unacceptable if CCC <0.5, poor if CCC = 0.5–0.6, mediocre if CCC = 0.6–0.7, satisfactory if CCC = 0.7–0.8, fairly good if CCC = 0.80–0.90, very good if CCC = 0.90–0.95, and excellent if CCC >0.95.^24^ Statistical analyses were performed using Prism, V5 (GraphPad, San Diego, CA), and R, V3.5.1 (R Foundation for Statistical Computing, Vienna, Austria).

## Results

### Clinical data

The study included 47 consecutive lymphedema patients (36 females) whose characteristics are shown in Table 1 and Supplementary Table S1.

**Table 1.** Descriptive Statistics of the Characteristics of the Population Sample

*n* = 47	Median [lower–upper quartile]	Mean ± SD	Range
Age (years)	59.0 [48.0–69.0]	58.3 ± 14.0	26.0–88.0
Height (m)	1.65 [1.60–1.71]	1.65 ± 0.09	1.45–1.92
Time with lymphedema (years)	5.0 [5.0–5.0]	4.7 ± 0.7	3.0–5.0
Body mass (kg) before treatment	82.1 [71.1–107.1]	87.9 ± 25.0	54.2–166.0
Body mass (kg) after treatment	81.9 [70.4–107.9]	86.8 ± 24.2	54.0–165.1

Results are provided as median [lower–upper quartile].

Thirty-two patients had primary lymphedema (68%) and 15 patients had secondary lymphedema (32%). Lymphedema had been diagnosed before 3 years of age in 4 patients, between 3 and 17 years of age in 6 patients, between 17 and 35 years of age in 8 patients, and after 35 years of age in 29 patients. Lymphedema involved 72 limbs: the right lower limb in 14 patients, the left in 8 patients, and both in 25 patients, leaving 22 unaffected limbs. Secondary lymphedema was cancer related in 13 patients and of mixed origin (chronic venous disease and obesity) in 2 patients. Lymphedema stage was 2A in 12 patients (18 limbs), 2B in 27 patients (41 limbs), and 3 in 8 patients (13 limbs).^16^ All patients completed the IDT session and underwent all ultrasonographic measurements.

### Comparison between lymphedema stages and normal limbs

The lower limb circumference was greater in the 72 limbs with lymphedema than in the 22 limbs without lymphedema at all levels before IDT and at all levels except the M1 level after IDT (Supplementary Table S1). Thickness of the epidermis, dermis, and subcutis was greater in the 72 limbs with lymphedema than in the 22 limbs without lymphedema before as well as after IDT, especially at the M3, M4, and M5 levels. There was no difference in elastic modulus between the 22 limbs without lymphedema and 72 limbs with lymphedema before IDT, except at the M5 level (Supplementary Table S2).

In patients with unilateral lymphedema, the circumference and dermis and subcutis thickness were greater at all levels except the M1 level, epidermis thickness was greater at the M4 and M5 levels, and elastic modulus was greater at the M5 level compared with the normal limb (Supplementary Table S3).

**FIG. 3.**
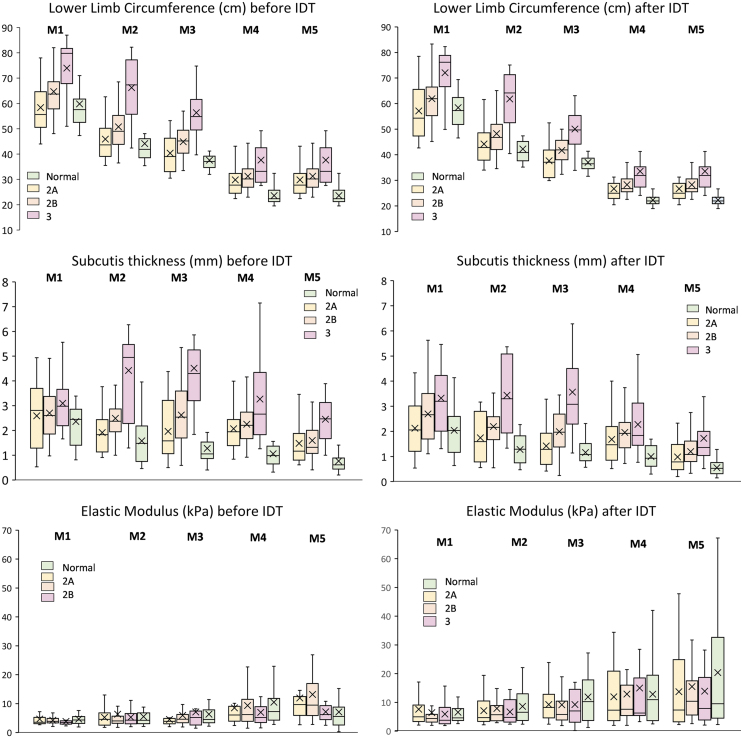
Box plots of lower limb circumference, subcutis thickness, and elastic modulus. Lower limb circumference, subcutis thickness, and elastic modulus at the five measurement levels (M1 to M5) before and after IDT for normal lower limbs and limbs with clinical stage 2A, 2B, and 3 lymphedema. The box upper and lower limits show the upper and lower quartiles. The line dividing the box is the median, and × shows the mean. The *whiskers* extend to the minimum and maximum values. IDT, intensive decongestive therapy.

Limb circumference and subcutis thickness were greater at all levels, before as well as after IDT, in the stage 3 group compared with the stage 2B and 2A lymphedema groups (Fig. 3). Epidermis or dermis thickness or elastic modulus differences between normal, stage 2A, stage 2B, and stage 3 lymphedema groups did not reach the significance level (Supplementary Table S4).

### Changes after decongestive therapy in lower limbs with lymphedema

There was a 0.4 [0–1.8] kg (*p* = 0.0003) decrease in body mass after IDT.

The volume of the affected limb was 10,980 [8458–13,960] mL before and 9607 [7720–11,830] mL after IDT (*p* < 0.0001). The decrease in lower limb volume was 1001 [650–2082] mL on the left side (*p* < 0.0001) and 1073 [617–2302] mL on the right side (*p* < 0.0001).

Circumferences of the affected lower limbs decreased (Table 2 and Supplementary Fig. S1) at all levels (*p* < 0.0001).

**Table 2.** Absolute Values and Relative Changes in Circumference, Skin Layer Thickness, and Subcutis Elastic Modulus in Lower Limbs with Lymphedema (*n* = 72) Before and After Intensive Decongestive Therapy at the Five Reference Levels

	M1	M2	M3	M4	M5
Limb circumference (cm)					
Before	63.65 [55.65 to 72.35]	49.40 [42.75 to 57.90]	45.25 [39.60 to 50.50]	29.85 [27.10 to 35.40]	29.85 [27.10 to 35.40]
After	62.00 [53.85 to 70.60]	47.40 [41.70 to 54.30]	41.85 [36.20 to 46.90]	27.35 [24.75 to 31.65]	27.35 [24.75 to 31.65]
Delta (%)	−3 [−6 to −2]	−4 [−7 to −3]	−7 [−10 to −4]	−9 [−13 to −4]	−9 [−13 to −4]
*p*	*p* **< 0.0001**	*p* **< 0.0001**	*p* **< 0.0001**	*p* **< 0.0001**	*p* **< 0.0001**
Epidermis thickness (mm)					
Before	0.07 [0.06 to 0.08]	0.07 [0.06 to 0.08]	0.07 [0.06 to 0.08]	0.07 [0.06 to 0.08]	0.07 [0.06 to 0.08]
After	0.07 [0.06 to 0.08]	0.07 [0.06 to 0.09]	0.07 [0.06 to 0.08]	0.07 [0.06 to 0.09]	0.07 [0.05 to 0.08]
Delta (%)	−5 [−0.25 to 18]	0 [−17 to 25]	0 [−17 to 25]	0 [−14 to 31]	0 [−21 to 20]
*p*	0.750	0.235	0.159	0.081	0.537
Dermis thickness (mm)					
Before	0.16 [0.14 to 0.20]	0.18 [0.15 to 0.24]	0.19 [0.14 to 0.26]	0.18 [0.14 to 0.28]	0.18 [0.12 to 0.23]
After	0.16 [0.13 to 0.21]	0.18 [0.14 to 0.24]	0.20 [0.16 to 0.25]	0.22 [0.15 to 0.28]	0.18 [0.14 to 0.23]
Delta (%)	−4 [−20 to 15]	−2 [−17 to 19]	0 [19 to 21]	0 [−25 to 48]	0 [−21 to 52]
*p*	0.833	0.943	0.393	0.246	0.052
Subcutis thickness (mm)					
Before	2.67 [1.82 to 3.51]	2.37 [1.77 to 3.11]	2.58 [1.58 to 3.75]	2.18 [1.68 to 2.91]	1.44 [1.07 to 2.29]
After	2.61 [1.70 to 3.51]	2.20 [1.46 to 2.90]	1.94 [1.29 to 2.82]	1.82 [1.33 to 2.36]	1.09 [0.75 to 1.68]
Delta (%)	−9 [−25 to 13]	−11 [−26 to 3]	−18 [−40 to −1]	−15 [−35 to 3]	−25 [−45 to −4]
*p*	0.253	**0.0011**	*p* **< 0.0001**	**0.0003**	*p* **< 0.0001**
Elastic modulus (kPa)					
Before	3.80 [3.15 to 4.80]	4.15 [3.20 to 5.80]	4.30 [3.25 to 6.30]	6.20 [3.85 to 10.15]	8.60 [5.65 to 12.80]
After	4.30 [3.15 to 7.50]	5.30 [3.40 to 9.90]	6.40 [4.10 to 11.05]	7.55 [5.15 to 16.70]	8.30 [4.60 to 18.45]
Delta (%)	13 [−21 to 90]	33 [−27 to 115]	40 [−13 to 169]	9 [−36 to 157]	−13 [−40 to 97]
*p*	**0.0039**	**0.0002**	*p* **< 0.0001**	**0.024**	0.093

*P* values are reported in bold characters when significant.

Results are provided as median [lower–upper quartile]; *p* = significance of comparison between values obtained before and after treatment by Wilcoxon signed-rank test.

IDT, intensive decongestive therapy; M1–M5, lower limb reference levels (as shown in Fig. 1).

There was no significant change in epidermis or dermis thickness after IDT, but a decrease in subcutis thickness at the M2 to M5 levels and an increase in elastic modulus at the M1 to M4 levels (Table 2 and Supplementary Fig. S1).

Neither changes in skin layer thickness nor changes in elastic modulus showed significant correlation with the relative change in limb volume (except for hypodermis thickness at the M5 level) (Supplementary Table S5). There was no significant correlation between IDT-induced changes in limb circumference and either elastic modulus before IDT or elastic modulus changes (Supplementary Table S6). Changes in subcutis thickness were significantly correlated with changes in elastic modulus at all limb levels (Supplementary Table S7).

### Reproducibility

Intraobserver reproducibility was fairly good at the M5 level and very good at all other levels for subcutis thickness measurement. It was unacceptable at the M1 and M4 levels, mediocre at the M2 and M5 levels, and fairly good at the M3 level for elastography (Supplementary Table S8). Interobserver reproducibility was unacceptable at all levels for elastography. Interobserver reproducibility of subcutis thickness measurement was unacceptable at the M1 and M2 levels, poor at the M4 level, and mediocre at the M3 and M5 levels (Supplementary Table S9).

## Discussion

The main findings of this study were as follows. (1) Limb circumference and dermis and subcutis thickness were greater in limbs with lymphedema than in limbs without lymphedema before as well as after IDT at most measurement levels, but the differences in elastic modulus did not reach the significance level. Limbs with stage 3 lymphedema had a greater circumference and thicker subcutis layer than stage 2A and 2B limbs, but there were no significant differences regarding other skin layers and elastic modulus.

(2) IDT produced a significant decrease not only in limb circumference and volume, but also in subcutis thickness, and an increase in elastic modulus. (3) Changes in elastic modulus correlated with changes in subcutis thickness, but not with limb circumference. (4) Intraobserver reproducibility was excellent for subcutis thickness measurement, but only fair to good for elastography. Interobserver reproducibility of elastography was unsatisfactory.

Lymphedema involves not only fluid accumulation but also adipose tissue remodeling and progressive fibrosis,^25^ altering tissue biomechanics. Lim et al. estimated skin and subcutaneous tissue stiffness by measuring the decrease in layer thickness induced by compression with the US probe in 39 upper limbs with postmastectomy lymphedema and reported lower stiffness in affected limbs.^26^

Kim et al. showed that this technique could differentiate normal from lymphedema limbs in 69 patients with breast cancer-related lymphedema.^27^ Suehiro et al. showed that skin and subcutis stiffness, evaluated by measuring tissue displacement induced by manual US probe compression, in 18 patients with lower limb lymphedema was lower in limbs with lymphedema than in unaffected limbs at the thigh (but not at the calf). After manual lymph drainage, these values increased at the calf (but not at the thigh). They concluded that manual lymph drainage seemed to “normalize” skin stiffness.^28^

US elastography provides automatic quantification of tissue stiffness. Greater tissue stiffness results in higher elastic modulus, thus higher SWV. Using acoustic radiation force impulse imaging (ARFI), Chan et al. found a significantly higher SWV in upper and lower limbs with lymphedema than in unaffected limbs.^29^ Erdogan et al. found a higher SWV at the forearm with stage 2 (*n* = 19), but not stage 1 (*n* = 17), lymphedema than at the unaffected forearm in patients with breast cancer-related lymphedema.^30^ Using ARFI, Bok et al. evaluated 45 upper limbs with postmastectomy lymphedema and reported a significant decrease in arm circumference and subcutaneous tissue thickness, and a decrease in SWV after pneumatic compression therapy, especially in the subgroup of patients treated with the higher compression pressure.^31^

Therefore, available elastography data are conflicting, which may be due not only to different population samples but also to technological and methodological issues. For instance, Erdogan et al. averaged the SWV measured in 10 different regions of interest (ROIs) disposed parallel to the skin surface,^30^ while Chan et al. measured the SWV in “many” 1 × 1-mm ROIs disposed perpendicular to the skin surface,^29^ and Bok et al. measured the SWV in three randomly placed 5 × 5-mm ROIs in the subcutis.^32^ In the present study, we performed elastography in a unique 4-mm-diameter ROI.

Regarding tissue elastic modulus, our results are in agreement with compliance measurements^26,27^ or elastography with manual or semistatic compression studies,^28,33^ which demonstrated lower tissue stiffness in limbs with lymphedema than in unaffected limbs and an increase in stiffness after IDT,^6,27,29,31,33^ but not with those of Bok et al. who reported decreased tissue stiffness. However, these authors did not provide detailed data (only changes and only mean values, but neither SD nor median and interquartile range).^31^

Besides technological differences, population sample characteristics, especially regarding the affected (upper or lower) limb, the etiology and stage of lymphedema, and the subjects' age, could explain some discrepancies, as suggested by Suehiro et al., who reported that skin strain in the lower range increased, whereas skin strain in the higher range decreased following manual lymphatic drainage, suggesting normalization.^6^

Hayashi et al. compared retrospective, qualitative elastography data and indocyanine green lymphography in 18 patients with lower limb lymphedema. They found a correlation between the size of the subcutaneous area and the lower stiffness and fluid accumulation shown by lymphography.^34^

Subcutaneous fluid collection is a common feature of lymphedema,^9,35^ resulting in spatial heterogeneity that may have contributed to the mediocre reproducibility of our elastographic measurements. Accumulated fluid may lead to greater tissue deformability.^26^

Thus, IDT, by evacuating fluid, may result in more homogeneous and stiff tissues. As elastography results are diverse, and as reproducibility, in our study, was unsatisfactory, technological and methodological issues should be overcome before elastography can be used in the clinical practice for lymphedema evaluation. Ultrasound backscattering is a different technique that may provide better characterization of lymphedema, as shown comparatively with lymphoscintigraphy by Lee et al. in 60 patients.^36^

Several authors used high-resolution B-mode US for measurement of skin and subcutis thickness in lymphedema.^3^ In 16 patients with lymphedema, 8 patients with lipedema, and 16 controls, Naouri et al. found significant differences in dermal thickness and observed that dermal hypoechogenicity was constant in limbs with lymphedema.^4^ Iker et al. also reported increased skin thickness and dermal hypoechogenicity in 10 patients with lower limb lymphedema and increased thickness and hypoechogenicity of the subcutaneous fat in 12 patients with lipedema.^5^

In 25 patients with postmastectomy lymphedema, Randheer et al. observed, after decongestive therapy, a parallel decrease in limb volume and skin and subcutis thickness.^8^ Suehiro et al. reported that skin and subcutis thickness and subcutaneous tissue echogenicity were positively correlated with the secondary lymphedema stage in 35 patients.^6^ Devoogdt et al. measured upper limb cutis and subcutis thickness in 42 patients immediately, 6 months, and 1 year after axillary dissection for breast cancer and observed greater subcutis thickness and disturbed cutis echogenicity in limbs developing lymphedema, but reported limited sensitivity and specificity.^9^

Using a 20-MHz ultrasound probe in 30 patients, Hacard et al. observed a 15% decrease in dermal thickness after IDT.^7^ Using a dedicated ultrasound system with a 20-MHz probe in patients with lymphedema, Phillips et al. reported excellent inter-rater reliability for skin thickness and echogenicity, although intersession reproducibility was challenging.^10^

Our results confirm the interest and reliability of skin layer thickness measurements by B-mode US, which demonstrated not only significant differences between affected and unaffected limbs but also a significant thickness decrease after IDT, especially for the subcutis layer. Limb circumference and subcutis thickness (especially at the M3, M4, and M5 levels) differentiated not only normal from lymphedema limbs but also stage 2A from stage 3 lymphedema before and after IDT, whereas thickness of the epidermis and dermis was different between normal and lymphedema limbs, but did not discriminate lymphedema stages.

The close correlation we found between relative IDT-induced changes in elastic modulus and in subcutis thickness suggests that fluid content was the main determinant of elastic modulus changes, overcoming lymphedema stage differences that could have been obscured by measurement variability. Although elastic modulus differences at baseline and IDT-induced changes did not always reach the significance level, they exhibited a clear pattern with higher values in unaffected limbs than in lymphedema limbs and after than before IDT, even in unaffected limbs. As the IDT regimen produced systemic effects also, a slight but significant decrease in unaffected limb circumference and subcutis thickness could be expected, with a subsequent decrease in elastic modulus, which is consistent with the present findings.

### Limitations

Although adequately powered for B-mode ultrasonographic measurement, our population sample was probably not large enough for elastography. However, the main issue regarding elastography appears to be its reproducibility, which needs specific technological and methodological improvements, including averaging multiple measurements within several larger ROIs. As we did not perform indocyanine green lymphography, we could not rely on objective data for lymphedema staging. Electric impedance measurement or dual-energy X-ray absorptiometry would help in investigating the relationship between elastic modulus and fluid content. Further multicentric studies of large series, including fully characterized lymphedema limbs, are still required.

## Conclusions

This study demonstrated that IDT induces a decrease in subcutis thickness and an increase in subcutis elastic modulus in lower limbs with lymphedema. However, elastographic measurements were hampered by poor reproducibility, emphasizing the need for specific technological and methodological improvement. Subcutis lymphatic fluid content appears to be the main determinant of tissue compliance and elastic modulus, at least with the ultrafast SWV imaging we used.

## Supplementary Material

Supplemental data
