# Supplementary material for: Skin Layer Thickness and Shear Wave Elastography Changes Induced by Intensive Decongestive Treatment of Lower Limb Lymphedema
Source: Lymphat Res Biol. 2022 Feb 28;20(1):17–25. doi: 10.1089/lrb.2021.0036 (PMC8892981; doi:10.1089/lrb.2021.0036)
Supplement: Supplemental data [file Suppl_Data.docx]

**Skin layer thickness and shear wave elastography changes induced by intensive decongestive treatment of lower limb lymphedema**

Merriem **Zarrad**, MD^1^, Sandrine **Mestre-Godin**, MD, PhD^1,2^, Gregory **Marin**, PhD^3^, M **Benhamou**, MD^1,2^, Jean-Pierre **Laroche**, MD^1^; Michel **Dauzat**, MD, PhD^4^; Claire **Duflos**, MD, PhD^3^, Isabelle **Quéré**, MD, PhD^1,2^

Table of contents

Supplemental Information: Ultrasonography 1

B-mode ultrasonography of skin 1

Shear wave elasticity imaging 1

Supplemental Figures 2

Supplemental Tables: 4

References 18

# Supplemental Information: Ultrasonography

## B-mode ultrasonography of skin

On B-mode ultrasonographic images, epidermis appears as the nearest hypoechoic line, while dermis presents as a hyperechoic band, and subcutis can be seen deeper as a hypoechoic layer segmented by hyperechoic longitudinal lines corresponding to fibrous septa.^(1)^

## Shear wave elasticity imaging

Shear wave elasticity imaging^(2)^ is achieved by focusing successively ultrasound beams at increasing depths, which create a source of acoustic radiation pressure that moves perpendicularly to the ultrasound beam at a speed higher than that of the shear waves it generates. This supersonic modality result in coherent summation of shear waves, increasing their amplitude and propagation distance. Then, ultrafast B-mode imaging (typically >5000 images per second) allows ultrasound speckle tracking of induced tissue deformation, and tissue elasticity, expressed as elastic modulus (in kPa), is derived from shear wave propagation velocity (SWV).^(3,4)^

# Supplemental Figures

**Supplemental Figure 1** – Difference between before and after treatment in limb circumference, subcutis thickness, and elastic modulus at the 5 references levels for normal lower limbs (n=22) and for limbs with lymphedema at stage 2A (n=18), 2B (n=41), and 3 (n=13).

|  |
| --- |

|  |
| --- |

|  |
| --- |

# Supplemental Tables:

**Supplemental Table I**-A– Individual characteristics of patients (first part)

| **Patient Number** | **Age (years)** | **Sex (M/F)** | **Height (m)** | **Side** | **Stage** | **Primary or Secondary** | **Etiology** | **History of dermo-hypodermitis** | **Age of occurrence** | **IDT duration (days)** |
| --- | --- | --- | --- | --- | --- | --- | --- | --- | --- | --- |
| 1 | 39 | F | 1.72 | R | 2A | Secondary | Cancer | Cancer | > 35 years | 3 |
| 2 | 70 | M | 1.77 | R | 2A | Primary |  | Spontaneous | > 35 years | 3 |
| 3 | 63 | M | 1.77 | L | 2B | Primary |  | Dermohypodermitis | > 35 years | 3 |
| 4 | 62 | F | 1.60 | L | 3 | Secondary | Cancer | Cancer | > 35 years | 3 |
| 5 | 66 | F | 1.65 | R | 2A | Primary |  | Spontaneous | < 3 years | 5 |
| 6 | 28 | M | 1.45 | L | 2B | Primary |  | Obesity | 17 - 35 years | 5 |
| 7 | 73 | F | 1.63 | B | 2B | Primary |  | Spontaneous | 3 - 16 years | 5 |
| 8 | 59 | F | 1.63 | R | 2B | Primary |  | traumatisme | > 35 years | 5 |
| 9 | 57 | F | 1.76 | L | 2B | Primary |  | Spontaneous | > 35 years | 4 |
| 10 | 46 | F | 1.63 | R | 2B | Primary |  | traumatisme | > 35 years | 5 |
| 11 | 66 | F | 1.5 | B | 2B | Primary |  | Spontaneous | > 35 years | 5 |
| 12 | 59 | F | 1.48 | B | 3 | Primary |  | Spontaneous | 17 - 35 years | 5 |
| 13 | 55 | F | 1.68 | B | 2A | Primary |  | Spontaneous | > 35 years | 3 |
| 14 | 74 | M | 1.81 | L | 2A | Secondary | Cancer | Cancer | > 35 years | 3 |
| 15 | 53 | F | 1.65 | L | 2B | Secondary | Cancer | Cancer | 17 - 35 years | 3 |
| 16 | 57 | F | 1.61 | L | 2A | Secondary | Cancer | Cancer | > 35 years | 5 |
| 17 | 36 | F | 1.62 | B | 2B | Primary |  | Spontaneous | 17 - 35 years | 5 |
| 18 | 64 | F | 1.68 | B | 3 | Secondary | incertaine | Dermohypodermitis | > 35 years | 5 |
| 19 | 61 | F | 1.71 | B | 2B | Primary |  | Spontaneous | 3 - 16 years | 5 |
| 20 | 69 | F | 1.55 | R | 3 | Secondary | Cancer | Cancer | > 35 years | 5 |
| 21 | 81 | F | 1.55 | R | 2A | Secondary | Cancer | Cancer | > 35 years | 5 |
| 22 | 59 | F | 1.6 | B | 2B | Primary |  | Spontaneous | 3 - 16 years | 5 |
| 23 | 40 | F | 1.75 | B | 2B | Primary |  | Spontaneous | < 3 years | 5 |
| 24 | 70 | F | 1.6 | B | 2B | Secondary | Cancer | Cancer | > 35 years | 5 |
| 25 | 51 | F | 1.63 | B | 2B | Secondary | Cancer | Cancer | 17 - 35 years | 5 |
| 26 | 67 | M | 1.62 | R | 2B | Primary |  | Spontaneous | > 35 years | 5 |
| 27 | 88 | F | 1.55 | B | 2A | Primary |  | Spontaneous | > 35 years | 5 |
| 28 | 76 | F | 1.65 | R | 2B | Secondary | Cancer | Cancer | > 35 years | 5 |
| 29 | 75 | M | 1.67 | B | 2A | Primary |  | Spontaneous | > 35 years | 5 |
| 30 | 47 | F | 1.52 | B | 2B | Primary |  | Spontaneous | 17 - 35 years | 5 |
| 31 | 56 | F | 1.66 | B | 2A | Secondary | incertaine | Spontaneous | > 35 years | 5 |
| 32 | 67 | F | 1.67 | R | 2B | Secondary | Cancer | Cancer | > 35 years | 5 |
| 33 | 59 | F | 1.6 | B | 2A | Primary |  | Spontaneous | > 35 years | 5 |
| 34 | 49 | F | 1.77 | R | 2B | Secondary | Cancer | Cancer | 17 - 35 years | 5 |
| 35 | 33 | M | 1.8 | B | 2B | Primary |  | Spontaneous | < 3 years | 5 |
| 36 | 72 | F | 1.68 | B | 2B | Primary |  | Spontaneous | > 35 years | 5 |
| 37 | 69 | F | 1.64 | B | 3 | Primary |  | Obesity | > 35 years | 5 |
| 38 | 26 | M | 1.71 | B | 2A | Primary |  | Spontaneous | 3 - 16 years | 5 |
| 39 | 61 | F | 1.58 | B | 2B | Primary |  | Spontaneous | > 35 years | 5 |
| 40 | 46 | M | 1.65 | B | 3 | Primary |  | Spontaneous | < 3 years | 5 |
| 41 | 54 | F | 1.6 | R | 2B | Primary |  | Spontaneous | > 35 years | 5 |
| 42 | 75 | M | 1.8 | L | 3 | Primary |  | traumatisme | > 35 years | 5 |
| 43 | 42 | F | 1.55 | B | 3 | Primary |  | Spontaneous | 17 - 35 years | 5 |
| 44 | 76 | M | 1.74 | B | 2B | Secondary | Cancer | Cancer | > 35 years | 5 |
| 45 | 53 | F | 1.7 | R | 2B | Primary |  | Spontaneous | > 35 years | 5 |
| 46 | 62 | F | 1.68 | B | 2B | Primary |  | Spontaneous | 3 - 16 years | 5 |
| 47 | 43 | F | 1.59 | R | 2B | Primary |  | Spontaneous | 3 - 16 years | 5 |

**Supplemental Table I**-B– Individual characteristics of patients (second part)

| **Patient Number** | **Pitting sign** | **Skin induration** | **Stemmer's sign** | **Fat deposition** | **Limb deformation** | **Lymphatic vesicles** | **Lympho-rrhea** | **Hyper-keratosis** | **Papillomas** | **Intertrigo** | **Varices** | **Pain** | **Skin color change** |
| --- | --- | --- | --- | --- | --- | --- | --- | --- | --- | --- | --- | --- | --- |
| 1 | Yes | No | No | No | No | No | No | Yes | No | No | No | No | No |
| 2 | Yes | No | No | No | No | No | No | No | No | No | No | No | No |
| 3 | Yes | Yes | Yes | No | No | Yes | No | No | No | No | Yes | No | No |
| 4 | Yes | Yes | Yes | No | No | Yes | No | No | No | Yes | No | No | No |
| 5 | No | No | No | No | No | No | No | No | No | No | No | No | No |
| 6 | Yes | No | No | Yes | Yes | No | No | No | No | Yes | No | No | Erythrosis |
| 7 | Yes | Yes | Yes | No | Yes | Yes | No | No | Yes | Yes | No | No | No |
| 8 | Yes | No | Yes | Yes | No | No | No | Yes | No | No | No | No | No |
| 9 | Yes | No | Yes | Yes | No | No | No | No | No | No | No | No | No |
| 10 | Yes | Yes | Yes | No | No | Yes | No | Yes | No | No | No | No | No |
| 11 | Yes | Yes | Yes | No | No | Yes | No | Yes | Yes | Yes | Yes | Yes | Yes |
| 12 | Yes | Yes | Yes | Yes | Yes | Yes | No | Yes | Yes | Yes | No | No | No |
| 13 | Yes | No | Yes | No | No | No | No | Yes | No | Yes | Yes | No | No |
| 14 | Yes | Yes | No | No | No | No | No | No | No | Yes | No | No | No |
| 15 | Yes | No | No | No | No | Yes | No | No | No | No | No | No | No |
| 16 | Yes | Yes | No | No | No | Yes | No | No | No | No | Yes | No | No |
| 17 | Yes | Yes | Yes | No | No | Yes | No | No | No | No | No | Yes | Yes |
| 18 | Yes | Yes | No | Yes | Yes | Yes | No | Yes | No | Yes | Yes | No | Hyper-pigmentation |
| 19 | Yes | Yes | Yes | No | No | Yes | No | Yes | No | Yes | Yes | No | Yes |
| 20 | Yes | Yes | Yes | Yes | Yes | Yes | Yes | Yes | No | Yes | No | Yes | No |
| 21 | Yes | Yes | No | No | No | No | No | No | No | Yes | Yes | Yes | Etythrosis |
| 22 | Yes | No | Yes | Yes | Yes | Yes | No | Yes | No | No | No | No | No |
| 23 | Yes | Yes | Yes | Yes | No | Yes | Yes | Yes | Yes | Yes | No | No | No |
| 24 | Yes | No | No | Yes | Yes | No | No | Yes | No | No | No | No | No |
| 25 | Yes | No | No | Yes | Yes | Yes | Yes | No | No | No | No | No | Erythema |
| 26 | Yes | Yes | No | Yes | Yes | Yes | Yes | No | No | Yes | No | No | No |
| 27 | Yes | Yes | No | Yes | No | No | No | Yes | No | Yes | No | No | No |
| 28 | Yes | Yes | Yes | Yes | Yes | Yes | Yes | Yes | No | Yes | No | No | No |
| 29 | Yes | No | No | No | No | No | No | No | No | Yes | Yes | No | Erythema |
| 30 | Yes | Yes | Yes | No | No | No | No | No | No | Yes | No | No | No |
| 31 | Yes | No | No | No | No | No | No | No | No | Yes | No | No | No |
| 32 | Yes | Yes | Yes | No | No | No | No | Yes | No | No | No | Yes | No |
| 33 | No | No | No | Yes | No | No | No | No | No | No | No | No | No |
| 34 | Yes | Yes | No | No | No | Yes | No | Yes | No | No | No | No | No |
| 35 | Yes | Yes | Yes | No | No | No | No | Yes | No | Yes | Yes | No | No |
| 36 | Yes | Yes | No | Yes | No | No | No | Yes | No | No | No | No | No |
| 37 | Yes | Yes | No | Yes | Yes | Yes | No | Yes | Yes | Yes | No | No | Yes |
| 38 | Yes | No | Yes | No | No | Yes | No | No | No | Yes | No | Yes | No |
| 39 | Yes | No | Yes | No | No | Yes | No | No | No | No | No | No | No |
| 40 | Yes | No | Yes | No | No | Yes | No | No | Yes | No | No | Yes | No |
| 41 | Yes | No | Yes | No | No | No | No | No | No | No | No | Yes | No |
| 42 | Yes | No | Yes | No | No | No | No | No | Yes | Yes | No | No | Erythema |
| 43 | Yes | No | Yes | No | No | No | No | Yes | No | Yes | No | No | Erythema |
| 44 | Yes | No | Yes | No | No | Yes | No | No | No | Yes | No | No | No |
| 45 | Yes | Yes | Yes | Yes | Yes | No | Yes | No | Yes | Yes | Yes | No | No |
| 46 | Yes | No | Yes | Yes | No | No | No | Yes | No | Yes | No | Yes | No |
| 47 | Yes | No | Yes | Yes | No | Yes | No | Yes | No | No | No | No | No |

**Supplemental Table II-A** – Data and comparison of the 22 lower limbs without and the 72 lower limbs with lymphedema before intensive decongestive therapy

| **Before IDT** | **Elasticity**  **(kPa)** | **Circumference**  **(cm)** | **Epidermis Thickness (mm)** | **Dermis Thickness**  **(mm)** | **SubCutis Thickness**  **(mm)** |
| --- | --- | --- | --- | --- | --- |
| **M1** | | | | | |
| Normal | 4.35 [3.10‒5.65] | 57.55 [52.50‒61.85] | 0.06 [0.05‒0.07] | 0.14 [0.12‒0.17] | 2.45 [1.39‒2.86] |
| Lymphedema | 3.80 [3.15‒4.80] | 63.65 [55.65‒72.35] | 0.07 [0.06‒0.08] | 0.16 [0.14‒0.20] | 2.67 [1.82‒3.51] |
| P | 0.1719 | **0.04** | **0.0018** | 0.0823 | 0.0924 |
| **M2** | | | | | |
| Normal | 4.25 [2.95‒7.20] | 41.85 [38.45‒46.20] | 0.06 [0.06‒0.08] | 0.16 [0.12‒0.18] | 1.48 [0.74‒2.19] |
| Lymphedema | 4.15 [3.20‒5.80] | 49.40 [42.75‒57.90] | 0.07 [0.06‒0.08] | 0.18 [0.15‒0.24] | 2.37 [1.77‒3.11] |
| P | 0.8303 | **0.0004** | 0.1575 | **0.025** | **0.0001** |
| **M3** | | | | | |
| Normal | 4.50 [3.25‒8.00] | 37.05 [34.70‒39.25] | 0.06 [0.05‒0.07] | 0.14 [0.12‒0.17] | 1.05 [0.86‒1.55] |
| Lymphedema | 4.30 [3.25‒6.30] | 45.25 [39.60‒50.50] | 0.07 [0.06‒0.08] | 0.19 [0.14‒0.26] | 2.58 [1.58‒3.75] |
| P | 0.5616 | **<0.0001** | **0.0109** | **0.0012** | **<0.0001** |
| **M4** | | | | | |
| Normal | 7.25 [4.20‒11.85] | 22.35 [21.20‒25.80] | 0.05 [0.04‒0.06] | 0.12 [0.09‒0.14] | 0.99 [0.64‒1.38] |
| Lymphedema | 6.20 [3.85‒10.15] | 29.85 [27.10‒35.40] | 0.07 [0.06‒0.08] | 0.18 [0.14‒0.28] | 2.18 [1.66‒2.84] |
| P | 0.4505 | **<0.0001** | **0.0011** | **<0.0001** | **<0.0001** |
| **M5** | | | | | |
| Normal | 5.55 [2.55‒8.90] | 22.35 [21.20‒25.80] | 0.05 [0.04‒0.06] | 0.12 [0.09‒0.14] | 0.61 [0.44‒0.92] |
| Lymphedema | 8.60 [5.65‒12.80] | 29.85 [27.10‒35.40] | 0.07 [0.06‒0.08] | 0.18 [0.12‒0.23] | 1.44 [1.07‒2.29] |
| P | **0.0133** | **<0.0001** | **<0.0001** | **0.0019** | **<0.0001** |

*Results are provided as median [lower – upper quartile]. P: P value of Mann-Whitney test for comparison between limbs without (n=22) and limbs with lymphedema (n=72).*

**Supplemental Table II-B** – Data and comparison of the 22 lower limbs without and the 72 lower limbs with lymphedema after intensive decongestive therapy

| **After IDT** | **Elasticity**  **(kPa)** | **Circumference**  **(cm)** | **Epidermis Thickness**  **(mm)** | **Dermis Thickness**  **(mm)** | **SubCutis Thickness**  **(mm)** |
| --- | --- | --- | --- | --- | --- |
| **M1** | | | | | |
| Normal | 4.60 [3.60‒9.10] | 57.30 [51.85‒62.40] | 0.06 [0.05‒0.07] | 0.13 [0.12‒0.16] | 2.04 [1.15‒2.62] |
| Lymphedema | 4.30 [3.15‒7.50] | 62.00 [53.85‒70.60] | 0.07 [0.06‒0.08] | 0.16 [0.13‒0.21] | 2.61 [1.70‒3.51] |
| P | 0.5467 | 0.112 | **0.0346** | **0.0283** | **0.0245** |
| **M2** | | | | | |
| Normal | 6.55 [3.55‒13.50] | 40.90 [37.65‒45.30] | 0.06 [0.05‒0.07] | 0.15 [0.12‒0.19] | 1.29 [0.73‒1.87] |
| Lymphedema | 5.30 [3.40‒9.90] | 47.40 [41.70‒54.30] | 0.07 [0.06‒0.09] | 0.18 [0.14‒0.24] | 2.20 [1.46‒2.90] |
| P | 0.4532 | **0.0013** | **0.0012** | **0.0139** | **<0.0001** |
| **M3** | | | | | |
| Normal | 10.25 [3.65‒18.70] | 36.50 [34.50‒38.75] | 0.06 [0.05‒0.06] | 0.15 [0.12‒0.19] | 1.08 [0.82‒1.52] |
| Lymphedema | 6.40 [4.10‒11.05] | 41.85 [36.20‒46.90] | 0.07 [0.06‒0.08] | 0.20 [0.16‒0.25] | 1.94 [1.29‒2.82] |
| P | 0.3889 | **0.0009** | **0.001** | **0.0004** | **0.0001** |
| **M4** | | | | | |
| Normal | 10.95 [3.60‒19.75] | 21.95 [20.75‒23.65] | 0.06 [0.05‒0.07] | 0.14 [0.12‒0.17] | 0.93 [0.61‒1.43] |
| Lymphedema | 7.55 [5.15‒16.70] | 27.35 [24.75‒31.65] | 0.07 [0.06‒0.09] | 0.22 [0.15‒0.28] | 1.82 [1.33‒2.36] |
| P | 0.9751 | **<0.0001** | **0.0053** | **0.0008** | **<0.0001** |
| **M5** | | | | | |
| Normal | 9.55 [4.25‒34.25] | 21.95 [20.75‒23.65] | 0.05 [0.04‒0.06] | 0.12 [0.09‒0.16] | 0.53 [0.31‒0.79] |
| Lymphedema | 8.30 [4.60‒18.45] | 27.35 [24.75‒31.65] | 0.07 [0.05‒0.08] | 0.18 [0.14‒0.23] | 1.09 [0.75‒1.68] |
| P | 0.4974 | **<0.0001** | **0.0022** | **0.0002** | **<0.0001** |

*Results are provided as median [lower – upper quartile]. P: P value of Mann-Whitney test for comparison between limbs without (n=22) and limbs with lymphedema (n=72).*

**Supplemental Table III –** Comparison between the affected (L) and the unaffected (N) lower limb in patients with unilateral lymphedema (n=22).

|  |  | **Lymphedema limbs** | | **Before vs after IDT** | **Normal limbs** | | **Before vs after IDT** | **L vs N** |
| --- | --- | --- | --- | --- | --- | --- | --- | --- |
|  |  | **Before IDT** | **Afer IDT** | **P** | **Before IDT** | **Afer IDT** | **P** | **P** |
|  | Limb Volume (ml) | 9353[8314‒12340] | 8576[5467‒9885] | **<0.0001** | 7891[6819‒8777] | 7699[6462‒8550] | **0.0002** | **0.0001** |
| **M1** | Circumference | 58.6[55.3‒64.7] | 55.4[53.3‒64.1] | **0.0003** | 57.6[52.5‒61.9] | 57.30 [51.85‒62.40] | 0.017 | 0.24 |
|  | Epidermis thickness | 0.07[0.06‒0.09] | 0.07[0.045‒0.085] | 0.72 | 0.055[0.045‒0.07] | 0.06 [0.05‒0.07] | 0.89 | **0.0025** |
|  | Dermis thickness | 0.17[0.14‒0.21] | 0.20[0.16‒0.23] | 0.045 | 0.14[0.12‒0.17] | 0.13 [0.12‒0.16] | 0.49 | 0.064 |
|  | Subcutis thickness | 2.44[1.81‒3.26] | 2.14[1.3‒3.06] | 0.081 | 2.45[1.39‒2.86] | 2.04 [1.15‒2.62] | 0.07 | 0.146 |
|  | Elastic Modulus | 3.85[2.8‒4.4] | 5.0[3.8‒9.05] | **0.0085** | 4.35[3.1‒5.65] | 4.60 [3.60‒9.10] | 0.13 | 0.125 |
| **M2** | Circumference | 46.2[42‒50.9] | 43.6[41.5‒48.4] | **<0.0001** | 41.9[38.5‒46.2] | 40.90 [37.65‒45.30] | **0.0014** | **<0.0001** |
|  | Epidermis thickness | 0.075[0.05‒0.09] | 0.075[0.06‒0.1] | 0.49 | 0.06[0.055‒0.075] | 0.06 [0.05‒0.07] | 0.64 | 0.395 |
|  | Dermis thickness | 0.19[0.14‒0.27] | 0.24[0.18‒0.28] | 0.98 | 0.16[0.12‒0.18] | 0.15 [0.12‒0.19] | 0.31 | **0.006** |
|  | Subcutis thickness | 2.27[1.38‒3.11] | 2.11[1.30‒2.86] | **0.006** | 1.48[0.74‒2.19] | 1.29 [0.73‒1.87] | **0.007** | **<0.0001** |
|  | Elastic Modulus | 4.85[3.2‒6.8] | 7.75[3.95‒12.1] | 0.028 | 4.25[2.95‒7.2] | 6.55 [3.55‒13.50] | **<0.0001** | 0.88 |
| **M3** | Circumference | 45.4[39.3‒50.2] | 42.7[37.5‒46.0] | **0.0002** | 37.1[34.7‒39.3] | 36.50 [34.50‒38.75] | **0.0022** | **0.0001** |
|  | Epidermis thickness | 0.07[0.05‒0.08] | 0.07[0.055‒0.08] | 0.94 | 0.06[0.05‒0.07] | 0.06 [0.05‒0.06] | 0.42 | 0.74 |
|  | Dermis thickness | 0.25[0.15‒0.30] | 0.21[0.19‒0.26] | 0.51 | 0.14[0.12‒0.17] | 0.15 [0.12‒0.19] | 0.53 | **0.002** |
|  | Subcutis thickness | 2.48[1.57‒3.78] | 1.63[1.23‒2.55] | **0.0006** | 1.05[0.86‒1.55] | 1.08 [0.82‒1.52] | 0.92 | **0.0007** |
|  | Elastic Modulus | 4.35[2.9‒6.0] | 8.6[3.65‒15.5] | **0.004** | 4.5[3.25‒8.0] | 10.25 [3.65‒18.70] | **0.003** | 0.215 |
| **M4** | Circumference | 27.9[26.0‒33.2] | 26.8[25.6‒31.2] | **0.0006** | 22.4[21.2‒25.8] | 21.95 [20.75‒23.65] | **0.004** | **0.0002** |
|  | Epidermis thickness | 0.07[0.06‒0.08] | 0.07[0.055‒0.09] | 0.79 | 0.05[0.04‒0.06] | 0.06 [0.05‒0.07] | 0.1 | **0.0005** |
|  | Dermis thickness | 0.225[0.155‒0.295] | 0.225[0.17‒0.305] | 0.99 | 0.12[0.09‒0.14] | 0.14 [0.12‒0.17] | 0.065 | **<0.0001** |
|  | Subcutis thickness | 2.02[1.64‒2.58] | 1.67[0.79‒2.325] | 0.01 | 0.99[0.64‒1.38] | 0.93 [0.61‒1.43] | 0.85 | **<0.0001** |
|  | Elastic Modulus | 4.7[3.3‒9.95] | 8.65[5.3‒20.9] | 0.029 | 7.25[4.2‒11.85] | 10.95 [3.60‒19.75] | 0.21 | 0.25 |
| **M5** | Circumference | 27.9[26.0‒33.2] | 26.8[24.6‒31.2] | **0.0006** | 22.4[21.2‒25.8] | 21.95 [20.75‒23.65] | **0.004** | **0.0002** |
|  | Epidermis thickness | 0.065[0.06‒0.08] | 0.07[0.045‒0.085] | 0.89 | 0.05[0.04‒0.06] | 0.05 [0.04‒0.06] | 0.41 | **0.0003** |
|  | Dermis thickness | 0.2[0.17‒0.25] | 0.19[0.14‒0.25] | 0.57 | 0.12[0.09‒0.14] | 0.12 [0.09‒0.16] | 0.79 | **0.0002** |
|  | Subcutis thickness | 1.25[0.86‒1.85] | 1.0[0.48‒1.45] | **0.002** | 0.61[0.44‒0.92] | 0.53 [0.31‒0.79] | 0.047 | **0.001** |
|  | Elastic Modulus | 7.7[5.4‒19.2] | 17.1[6.6‒27.2] | 0.13 | 5.55[2.55‒8.9] | 9.55 [4.25‒34.25] | **0.004** | 0.032 |

*L vs N: P value of Wilcoxon-Mann-Whitney test for comparison of affected and unaffected limbs. Before vs after IDT: P value of Wilcoxon signed-rank test.*

**Supplemental Table IV-A** – Elasticity in limbs without lymphedema (n=22), and with stage 2A (n=18), stage 2B (n=41), and stage 3 (n=13) lymphedema, before and after intensive decongestion therapy.

| **Elasticity**  **(kPa)** | **2A** | **2B** | **3** | **N** | **Kruskal-Wallis P** | **N vs 2A** | **N vs 2B** | **N vs 3** | **2A vs 2B** | **2A vs 3** | **2B vs 3** |
| --- | --- | --- | --- | --- | --- | --- | --- | --- | --- | --- | --- |
| M1 Before | 3.55 [3.05‒5.25] | 3.90 [3.35‒4.90] | 3.50 [2.90‒4.05] | 4.35 [3.10‒5.65] | 0.3293 |  |  |  |  |  |  |
| After | 4.95 [3.20‒9.15] | 4.40 [3.15‒5.85] | 3.80 [2.55‒8.25] | 4.60 [3.60‒9.10] | 0.6669 |  |  |  |  |  |  |
| M2 Before | 4.55 [2.55‒6.95] | 4.00 [3.20‒5.65] | 4.30 [3.00‒6.55] | 4.25 [2.95‒7.20] | 0.9924 |  |  |  |  |  |  |
| After | 4.70 [3.35‒10.55] | 5.70 [3.60‒8.90] | 4.80 [3.15‒10.95] | 6.55 [3.55‒13.50] | 0.7809 |  |  |  |  |  |  |
| M3 Before | 3.90 [2.90‒4.95] | 4.60 [3.40‒6.25] | 5.20 [2.60‒7.45] | 4.50 [3.25‒8.00] | 0.6114 |  |  |  |  |  |  |
| After | 8.25 [4.55‒12.85] | 5.80 [3.90‒10.45] | 7.00 [3.10‒14.55] | 10.25 [3.65‒18.70] | 0.6568 |  |  |  |  |  |  |
| M4 Before | 6.05 [3.85‒9.25] | 6.20 [3.90‒11.50] | 5.20 [3.45‒8.95] | 7.25 [4.20‒11.85] | 0.6122 |  |  |  |  |  |  |
| After | 7.30 [3.60‒21.35] | 7.60 [5.45‒16.00] | 6.30 [5.45‒18.50] | 10.95 [3.60‒19.75] | 0.9605 |  |  |  |  |  |  |
| M5 Before | 9.65 [5.90‒13.15] | 9.50 [5.90‒16.95] | 6.20 [4.45‒9.30] | 5.55 [2.55‒8.90] | **0.0113** |  | ***** |  |  |  |  |
| After | 7.35 [3.20‒26.00] | 10.40 [5.60‒17.50] | 7.90 [3.90‒18.70] | 9.55 [4.25‒34.25] | 0.7452 |  |  |  |  |  |  |

**Supplemental Table III-B** – Circumferences of limbs without (n=22), and with stage 2A (n=18), stage 2B (n=41), and stage 3 (n=13) lymphedema.

| **Circumference (cm)** | **L2A** | **L2B** | **L3** | **N** | **Kruskal-Wallis P** | **N vs 2A** | **N vs 2B** | **N vs 3** | **2A vs 2B** | **2A vs 3** | **2B vs 3** |
| --- | --- | --- | --- | --- | --- | --- | --- | --- | --- | --- | --- |
| M1 Before | 55.65 [49.80‒65.70] | 63.70 [57.85‒68.55] | 79.80 [67.85‒81.70] | 57.55 [52.50‒61.85] | **0.0006** |  |  | ** |  | ** |  |
| After | 54.35 [47.35‒67.15] | 61.90 [55.30‒66.50] | 76.20 [66.65‒78.85] | 57.30 [51.85‒62.40] | **0.0014** |  |  | ** |  | ** |  |
| M2 Before | 43.65 [38.80‒50.45] | 49.00 [43.85‒55.25] | 67.30 [55.75‒77.20] | 41.85 [38.45‒46.20] | **<0.0001** |  | * | *** |  | *** | * |
| After | 42.85 [37.60‒48.75] | 46.80 [42.05‒51.90] | 64.20 [52.55‒71.30] | 40.90 [37.65‒45.30] | **<0.0001** |  | * | *** |  | ** | * |
| M3 Before | 39.00 [33.00‒47.15] | 45.20 [40.35‒49.40] | 54.80 [49.55‒61.60] | 37.05 [34.70‒39.25] | **<0.0001** |  | *** | *** |  | *** | * |
| After | 37.10 [31.05‒41.90] | 41.90 [38.05‒45.65] | 49.70 [44.15‒55.35] | 36.50 [34.50‒38.75] | **<0.0001** |  | ** | *** |  | *** |  |
| M4 Before | 27.75 [24.45‒35.40] | 30.20 [26.90‒34.15] | 33.20 [28.90‒42.50] | 22.35 [21.20‒25.80] | **<0.0001** | * | *** | *** |  |  |  |
| After | 25.00 [22.65‒29.50] | 26.80 [25.55‒30.60] | 31.90 [27.40‒35.30] | 21.95 [20.75‒23.65] | **<0.0001** | * | *** | *** |  | * |  |
| M5 Before | 9.65 [5.90‒13.15] | 9.50 [5.90‒16.95] | 6.20 [4.45‒9.30] | 5.55 [2.55‒8.90] | **0.0113** |  | * |  |  |  |  |
| After | 7.35 [3.20‒26.00] | 10.40 [5.60‒17.50] | 7.90 [3.90‒18.70] | 9.55 [4.25‒34.25] | 0.7452 |  |  |  |  |  |  |

* P<.05 ; ** P<.01 ; *** P<.001

**Supplemental Table IV-C** – Epidermis thickness in limbs without (n=22), and with stage 2A (n=18), stage 2B (n=41), and stage 3 (n=13) lymphedema.

| **Epidermis thickness**  **(mm)** | **L2A** | **L2B** | **L3** | **N** | **Kruskal-Wallis P** | **N vs 2A** | **N vs 2B** | **N vs 3** | **2A vs 2B** | **2A vs 3** | **2B vs 3** |
| --- | --- | --- | --- | --- | --- | --- | --- | --- | --- | --- | --- |
| M1 Before | 0.08 [0.06‒0.09] | 0.07 [0.06‒0.08] | 0.07 [0.07‒0.08] | 0.06 [0.05‒0.07] | **0.0173** | * | * |  |  |  |  |
| After | 0.06 [0.04‒0.08] | 0.07 [0.06‒0.08] | 0.07 [0.06‒0.08] | 0.06 [0.05‒0.07] | 0.0869 |  |  |  |  |  |  |
| M2 Before | 0.07 [0.05‒0.09] | 0.08 [0.06‒0.08] | 0.07 [0.06‒0.08] | 0.06 [0.06‒0.08] | 0.2285 |  |  |  |  |  |  |
| After | 0.07 [0.05‒0.07] | 0.07 [0.06‒0.10] | 0.08 [0.07‒0.08] | 0.06 [0.05‒0.07] | **0.0013** |  | ** | * |  |  |  |
| M3 Before | 0.06 [0.06‒0.08] | 0.07 [0.06‒0.08] | 0.08 [0.06‒0.10] | 0.06 [0.05‒0.07] | **0.0252** |  |  | * |  |  |  |
| After | 0.07 [0.06‒0.09] | 0.07 [0.06‒0.08] | 0.07 [0.06‒0.10] | 0.06 [0.05‒0.06] | **0.0099** |  | * | * |  |  |  |
| M4 Before | 0.07 [0.06‒0.07] | 0.07 [0.06‒0.08] | 0.07 [0.04‒0.09] | 0.05 [0.04‒0.06] | **0.0089** |  | ** |  | * |  |  |
| After | 0.06 [0.05‒0.07] | 0.08 [0.07‒0.09] | 0.07 [0.05‒0.10] | 0.06 [0.05‒0.07] | **0.0008** |  | ** |  |  |  |  |
| M5 Before | 0.06 [0.06‒0.08] | 0.07 [0.06‒0.08] | 0.06 [0.04‒0.08] | 0.05 [0.04‒0.06] | **0.0004** | * | *** |  |  |  |  |
| After | 0.07 [0.04‒0.08] | 0.07 [0.05‒0.08] | 0.06 [0.05‒0.08] | 0.05 [0.04‒0.06] | **0.019** |  | * |  |  |  |  |

**Supplemental Table IV-D** – Dermis thickness in limbs without (n=22), and with stage 2A (n=18), stage 2B (n=41), and stage 3 (n=13) lymphedema.

| **Dermis thickness (mm)** | **L2A** | **L2B** | **L3** | **N** | **Kruskal-Wallis P** | **N vs 2A** | **N vs 2B** | **N vs 3** | **2A vs 2B** | **2A vs 3** | **2B vs 3** |
| --- | --- | --- | --- | --- | --- | --- | --- | --- | --- | --- | --- |
| M1 Before | 0.16 [0.15‒0.19] | 0.15 [0.13‒0.21] | 0.17 [0.16‒0.22] | 0.14 [0.12‒0.17] | **0.019** |  | * |  |  |  |  |
| After | 0.16 [0.13‒0.20] | 0.16 [0.13‒0.21] | 0.19 [0.15‒0.24] | 0.13 [0.12‒0.16] | 0.0507 |  |  |  |  |  |  |
| M2 Before | 0.18 [0.15‒0.23] | 0.18 [0.14‒0.25] | 0.18 [0.15‒0.26] | 0.16 [0.12‒0.18] | 0.1639 |  |  |  |  |  |  |
| After | 0.16 [0.13‒0.19] | 0.20 [0.15‒0.26] | 0.20 [0.15‒0.29] | 0.15 [0.12‒0.19] | **0.0063** |  | * |  |  |  |  |
| M3 Before | 0.19 [0.14‒0.25] | 0.18 [0.14‒0.27] | 0.20 [0.14‒0.26] | 0.14 [0.12‒0.17] | **0.0136** |  | * |  |  |  |  |
| After | 0.18 [0.15‒0.21] | 0.21 [0.15‒0.25] | 0.20 [0.17‒0.25] | 0.15 [0.12‒0.19] | **0.0032** |  | ** | * |  |  |  |
| M4 Before | 0.20 [0.16‒0.30] | 0.18 [0.13‒0.28] | 0.15 [0.08‒0.23] | 0.12 [0.09‒0.14] | **<0.0001** | *** | ** |  |  |  |  |
| After | 0.20 [0.13‒0.23] | 0.23 [0.16‒0.30] | 0.20 [0.11‒0.27] | 0.14 [0.12‒0.17] | **0.0028** |  | ** |  |  |  |  |
| M5 Before | 0.17 [0.14‒0.24] | 0.20 [0.12‒0.23] | 0.13 [0.07‒0.24] | 0.12 [0.09‒0.14] | **0.007** | * | ** |  |  |  |  |
| After | 0.19 [0.13‒0.23] | 0.18 [0.15‒0.23] | 0.16 [0.11‒0.31] | 0.12 [0.09‒0.16] | **0.0028** |  | ** |  |  |  |  |

* P<.05 ; ** P<.01 ; *** P<.001

**Supplemental Table IV-E** – Subcutis thickness in limbs without (n=22), and with stage 2A (n=18), stage 2B (n=41), and stage 2 (n=13) lymphedema.

| **Subcutis** | **L2A** | **L2B** | **L3** | **N** | **Kruskal-Wallis P** | **N vs 2A** | **N vs 2B** | **N vs 3** | **2A vs 2B** | **2A vs 3** | **2B vs 3** |
| --- | --- | --- | --- | --- | --- | --- | --- | --- | --- | --- | --- |
| M1 Before | 2.81 [1.24‒3.78] | 2.60 [1.86‒3.38] | 2.98 [2.19‒3.66] | 2.45 [1.39‒2.86] | 0.2658 |  |  |  |  |  |  |
| After | 2.06 [1.18‒3.02] | 2.66 [1.70‒3.51] | 3.20 [2.01‒4.23] | 2.04 [1.15‒2.62] | **0.0081** |  |  | * |  |  |  |
| M2 Before | 1.83 [1.12‒2.50] | 2.37 [1.95‒2.87] | 4.95 [2.28‒5.47] | 1.48 [0.74‒2.19] | **<0.0001** |  | ** | *** |  | ** |  |
| After | 1.60 [0.77‒2.83] | 2.16 [1.67‒2.59] | 3.30 [1.94‒5.08] | 1.29 [0.73‒1.87] | **<0.0001** |  | *** | *** |  | * |  |
| M3 Before | 1.58 [1.06‒3.24] | 2.54 [1.70‒3.59] | 4.30 [3.21‒5.26] | 1.05 [0.86‒1.55] | **<0.0001** |  | *** | *** |  | ** | * |
| After | 1.29 [0.68‒2.00] | 1.97 [1.38‒2.69] | 3.08 [2.29‒4.50] | 1.08 [0.82‒1.52] | **<0.0001** |  | ** | *** |  | *** |  |
| M4 Before | 1.95 [1.33‒2.47] | 2.21 [1.67‒2.74] | 2.66 [1.91‒4.24] | 0.99 [0.64‒1.38] | **<0.0001** | ** | *** | *** |  |  |  |
| After | 1.47 [0.84‒2.30] | 1.92 [1.35‒2.36] | 1.84 [1.45‒3.13] | 0.93 [0.61‒1.43] | **<0.0001** | * | *** | *** |  | * |  |
| M5 Before | 1.17 [0.78‒1.92] | 1.32 [1.08‒2.01] | 2.47 [1.67‒3.13] | 0.61 [0.44‒0.92] | **<0.0001** | * | *** | *** |  | * |  |
| After | 0.79 [0.48‒1.55] | 1.08 [0.80‒1.62] | 1.36 [1.04‒2.00] | 0.53 [0.31‒0.79] | **<0.0001** |  | *** | *** |  |  |  |

* P<.05 ; ** P<.01 ; *** P<.001

**Supplemental Table V – Correlation between relative changes in ultrasonographic measurements and relatives changes in limb volume (n=72).**

| **Volume Delta % *versus*** | **Epidermis thickness Delta%** | **Dermis thickness Delta%** | **Subcutis thickness Delta%** | **Elastic Modulus Delta%** |
| --- | --- | --- | --- | --- |
| **M1** | | | | |
| **Spearman r** | 0.0472 | 0.1338 | -0.0124 | 0.0570 |
| **P value (two-tailed)** | 0.6939 | 0.2624 | 0.918 | 0.6346 |
| **r²** | 0.0018 | 0.0286 | 0.0015 | 0.00005 |
| **P value** | 0.7232 | 0.1558 | 0.748 | 0.954 |
| **M2** | | | | |
| **Spearman r** | 0.09819 | 0.1734 | 0.04994 | -0.03084 |
| **P value (two-tailed)** | 0.4119 | 0.1452 | 0.677 | 0.7971 |
| **r²** | 0.0208 | 0.0005 | 0.0088 | 0.0028 |
| **P value** | 0.227 | 0.8498 | 0.4328 | 0.6612 |
| **M3** | | | | |
| **Spearman r** | -0.07549 | -0.003377 | 0.2972 | 0.0379 |
| **P value (two-tailed)** | 0.5285 | 0.9775 | **0.0112** | 0.752 |
| **r²** | 0.0105 | 0.0025 | 0.0056 | 0.0052 |
| **P value** | 0.3929 | 0.6754 | 0.532 | 0.5478 |
| **M4** | | | | |
| **Spearman r** | 0.03162 | 0.2028 | 0.0191 | 0.06423 |
| **P value (two-tailed)** | 0.7921 | 0.0875 | 0.8735 | 0.5919 |
| **r²** | 0.0001 | 0.0114 | 0.0006 | 0.00004 |
| **P value** | 0.9352 | 0.3718 | 0.8429 | 0.9555 |
| **M5** | | | | |
| **Spearman r** | 0.0174 | 0.1199 | 0.2813 | 0.08753 |
| **P value (two-tailed)** | 0.8846 | 0.3158 | **0.0167** | 0.4647 |
| **r²** | 0.0003 | 0.0096 | 0.0659 | 0.0150 |
| **P value** | 0.8892 | 0.412 | **0.0295** | 0.305 |

*Legend: Spearman r: correlation coefficient and linear regression; P value (two-tailed): signification of Spearman correlation test. r2 and P value: results of linear correlation analysis.*

**Supplemental Table VI – Correlation between circumference changes and elastic modulus values before treatment or elastic modulus change in lower limbs with lymphedema (n=72).**

| **Circumference Delta**  ***versus*:** | **M1** | **M2** | **M3** | **M4** | **M5** |
| --- | --- | --- | --- | --- | --- |
| **Elastic Modulus change** | | | | | |
| Spearman r | 0.02852 | -0.1664 | 0.1023 | 0.1412 | 0.2368 |
| P value (two-tailed) | 0.8120 | 0.1625 | 0.3926 | 0.2368 | 0.2573 |
| r^2^ | 0.0003322 | 0.04606 | 0.03615 | 0.01154 | 0.01773 |
| P | 0.8792 | 0.0702 | 0.1096 | 0.3691 | 0.2649 |
| **Elastic Modulus before treatment** | | | | | |
| Spearman r | -0.07345 | -0.1664 | -0.2408 | 0.04655 | 0.002051 |
| P value (two-tailed) | 0.5397 | 0.1625 | **0.0416** | 0.6978 | 0.9864 |
| r^2^ | 0.00009908 | 0.04606 | 0.006713 | 0.0000004932 | 0.006591 |
| P | 0.9339 | 0.0702 | 0.4938 | 0.9953 | 0.4978 |

**Supplemental Table VII - Correlation between relative changes in elastic modulus and relative changes in epidermis, dermis, and hypodermis thickness (n=72).**

| **Elastography Delta % versus** | **Epidermis thickness Delta%** | **Dermis thickness Delta%** | **Subcutis thickness Delta%** |
| --- | --- | --- | --- |
| **M1** | | | |
| **Spearman r** | -0.0656 | -0.003 | -0.204 |
| **P value (two-tailed)** | 0.588 | 0.980 | 0.086 |
| **r²** | 0.0003 | 0.0002 | 0.1134 |
| **P value** | 0.8842 | 0.9178 | **0.0038** |
| **M2** | | | |
| **Spearman r** | -0.0235 | 0.0524 | -0.5891 |
| **P value (two-tailed)** | 0.845 | 0.6622 | **<0.0001** |
| **r²** | 0.0047 | 0.0130 | 0.2342 |
| **P value** | 0.5674 | 0.3402 | **< 0.0001** |
| **M3** | | | |
| **Spearman r** | -0.0926 | -0.1277 | -0.5371 |
| **P value (two-tailed)** | 0.4394 | 0.2849 | **<0.0001** |
| **r²** | 0.0166 | 0.00036 | 0.1661 |
| **P value** | 0.2813 | 0.8752 | **0.0004** |
| **M4** | | | |
| **Spearman r** | -0.0187 | 0.0830 | -0.2715 |
| **P value (two-tailed)** | 0.8759 | 0.4882 | **0.0211** |
| **r²** | 0.0005 | 0.0624 | 0.1117 |
| **P value** | 0.8518 | **0.0343** | **0.0041** |
| **M5** | | | |
| **Spearman r** | 0.0174 | 0.1199 | 0.2813 |
| **P value (two-tailed)** | 0.8846 | 0.3158 | **0.0167** |
| **r²** | 0.0003 | 0.0096 | 0.0659 |
| **P value** | 0.8892 | 0.412 | **0.0295** |

*Legend: Spearman r: correlation coefficient and linear regression; P value (two-tailed): signification of Spearman correlation test. r2 and P value: results of linear correlation analysis.*

**Supplemental Table VIII – Intra-observer reproducibility (n=30)**

|  | **M1** | **M2** | **M3** | **M4** | **M5** | **All sites** |
| --- | --- | --- | --- | --- | --- | --- |
| **Elastic Modulus** | | | | | | |
| **ICC** | 0.4665 | 0.6875 | 0.8419 | 0.3698 | 0.6654 | 0.6779 |
| **lower** | 0.1433 | 0.4445 | 0.6962 | 0.0094 | 0.4032 | 0.5809 |
| **upper** | 0.7025 | 0.8371 | 0.9213 | 0.6428 | 0.8260 | 0.7559 |
| **Subcutis Thickness** | | | | | | |
| **ICC** | 0.9368 | 0.9497 | 0.9361 | 0.9461 | 0.8765 | 0.9466 |
| **lower** | 0.8727 | 0.8981 | 0.8704 | 0.8893 | 0.7565 | 0.9268 |
| **upper** | 0.9693 | 0.9757 | 0.9690 | 0.9741 | 0.9393 | 0.9612 |

*Legend: Intraclass correlation coefficient (ICC type 1, 2 classes, at alpha 0.05) for intra-observer reproducibility in 30 patients.*

**Supplemental Table IX – Inter-observer reproducibility (n=30)**

|  | **M1** | **M2** | **M3** | **M4** | **M5** | **All sites** |
| --- | --- | --- | --- | --- | --- | --- |
| **Elastic Modulus** | | | | | | |
| **ICC** | 0.0431 | 0.0212 | 0.0893 | 0.0269 | 0.0018 | 0.0853 |
| **lower** | -0.1030 | -0.0584 | -0.0801 | -0.1122 | -0.1465 | -0.0443 |
| **upper** | 0.2348 | 0.1432 | 0.3019 | 0.2130 | 0.1979 | 0.2184 |
| **Sub Cutaneous Thickness** | | | | | | |
| **ICC** | 0.2718 | 0.3426 | 0.6121 | 0.5483 | 0.6803 | 0.5075 |
| **lower** | -0.1097 | -0.0863 | 0.0861 | 0.2607 | 0.4702 | 0.0929 |
| **upper** | 0.6198 | 0.6641 | 0.8284 | 0.7402 | 0.8173 | 0.7211 |

# References

1. Barcaui, Ede O., Carvalho, A. C., Lopes, F. P., Pineiro-Maceira, J., and Barcaui, C. B. *High frequency ultrasound with color Doppler in dermatology*. **An.Bras.Dermatol.** 2016;91:262-273.

2. Sarvazyan, A. P., Rudenko, O. V., Swanson, S. D., Fowlkes, J. B., and Emelianov, S. Y. *Shear wave elasticity imaging: a new ultrasonic technology of medical diagnostics*. **Ultrasound Med Biol** 1998;24:1419-1435.

3. Gennisson, J. L., Renier, M., Catheline, S., Barriere, C., Bercoff, J., Tanter, M., and Fink, M. *Acoustoelasticity in soft solids: assessment of the nonlinear shear modulus with the acoustic radiation force*. **J Acoust.Soc.Am.** 2007;122:3211-3219.

4. Bercoff, J., Tanter, M., and Fink, M. *Supersonic shear imaging: a new technique for soft tissue elasticity mapping*. **IEEE Trans.Ultrason.Ferroelectr.Freq.Control** 2004;51:396-409.

1. Sarvazyan, A. P., Rudenko, O. V., Swanson, S. D., Fowlkes, J. B., and Emelianov, S. Y. *Shear wave elasticity imaging: a new ultrasonic technology of medical diagnostics*. **Ultrasound Med Biol** 1998;24:1419-1435.

2. Gennisson, J. L., Renier, M., Catheline, S., Barriere, C., Bercoff, J., Tanter, M., and Fink, M. *Acoustoelasticity in soft solids: assessment of the nonlinear shear modulus with the acoustic radiation force*. **J Acoust.Soc.Am.** 2007;122:3211-3219.

3. Bercoff, J., Tanter, M., and Fink, M. *Supersonic shear imaging: a new technique for soft tissue elasticity mapping*. **IEEE Trans.Ultrason.Ferroelectr.Freq.Control** 2004;51:396-409.
